# Body Size Predicts Cardiac and Vascular Resistance Effects on Men's and Women's Blood Pressure

**DOI:** 10.3389/fphys.2017.00561

**Published:** 2017-08-09

**Authors:** Joyce M. Evans, Siqi Wang, Christopher Greb, Vladimir Kostas, Charles F. Knapp, Qingguang Zhang, Eric S. Roemmele, Michael B. Stenger, David C. Randall

**Affiliations:** ^1^Department of Biomedical Engineering, University of Kentucky Lexington, KY, United States; ^2^Department of Statistics, University of Kentucky Lexington, KY, United States; ^3^Wyle Science, Technology and Engineering Group Houston, TX, United States; ^4^Department of Physiology, University of Kentucky Lexington, KY, United States

**Keywords:** orthostatic, stroke volume, cardiac output, total peripheral resistance, blood pressure, body size

## Abstract

**Key Points Summary**

- We report how blood pressure, cardiac output and vascular resistance are related to height, weight, body surface area (BSA), and body mass index (BMI) in healthy young adults at supine rest and standing.
- Much inter-subject variability in young adult's blood pressure, currently attributed to health status, may actually result from inter-individual body size differences.
- Each cardiovascular variable is linearly related to height, weight and/or BSA (more than to BMI).
- When supine, cardiac output is positively related, while vascular resistance is negatively related, to body size. Upon standing, the change in vascular resistance is positively related to size.
- The height/weight relationships of cardiac output and vascular resistance to body size are responsible for blood pressure relationships to body size.
- These basic components of blood pressure could help distinguish normal from abnormal blood pressures in young adults by providing a more effective scaling mechanism.

**Introduction:** Effects of body size on inter-subject blood pressure (BP) variability are not well established in adults. We hypothesized that relationships linking stroke volume (SV), cardiac output (CO), and total peripheral resistance (TPR) with body size would account for a significant fraction of inter-subject BP variability.

**Methods:** Thirty-four young, healthy adults (19 men, 15 women) participated in 38 stand tests during which brachial artery BP, heart rate, SV, CO, TPR, and indexes of body size were measured/calculated.

**Results:** Steady state diastolic arterial BP was not significantly correlated with any index of body size when subjects were supine. However, upon standing, the more the subject weighed, or the taller s/he was, the greater the increase in diastolic pressure. Systolic pressure strongly correlated with body weight and height both supine and standing. Diastolic and systolic BP were more strongly related to height, weight and body surface area than to body mass index. When supine: lack of correlation between diastolic pressure and body size, resulted from the combination of positive SV correlation and negative TPR correlation with body size. The positive systolic pressure vs. body size relationship resulted from a positive SV vs. height relationship. In response to standing: the positive diastolic blood pressure vs. body size relationship resulted from the standing-induced, positive increase in TPR vs. body size relationship. The relationships between body weight or height with SV and TPR contribute new insight into mechanisms of BP regulation that may aid in the prediction of health in young adults by providing a more effective way to scale BP with body size.

## Introduction

The scaling, or normalization, of cardiovascular variables in patients, especially arterial blood pressure (BP) is critically important in the evaluation of cardiac pathologies, and a review of prior work points out the necessity for seeking further explanation of inter-individual cardiovascular variability in healthy adults (Dewey et al., 2008; Joyner et al., 2015). In particular, Dewey et al. (2008) describe how appropriate morphometric scaling of cardiac variables could help to reduce inter-individual variability in the “normal” adult population and thereby provide more accurate discrimination of pathological changes. They illustrate this by contrasting the futility of discriminating cardiomyopathy from training effects in the “healthy” population where height ranges from 160 (average Chinese woman in 2001) to 201 cm (NBA player for the 2006–2007 season) and corresponding weight ranges from 47 to 101 kg. Joyner et al speculate that inter-individual variability in physiological data, particularly cardiac output, peripheral resistance and muscle sympathetic nerve activity, might be lowered with the application of judicious scaling (Joyner et al., 2015). Unfortunately, the dependence of BP and its cardiac output (CO) and total peripheral resistance (TPR) components upon body size are relatively obscure in adult, human physiological literature.

Large, epidemiologic studies have established BP relationships related to age, gender and ethnicity (Whitlock et al., 2009; Wright et al., 2011; Hosseini et al., 2015). Two studies determined an essentially linear relationship between systolic blood pressure (SBP) and age, with greater values (~8 mmHg, normal men and ~32 mmHg, untreated hypertensive women) in oldest vs. youngest subjects (ages 18–83 years) (Wright et al., 2011; Hosseini et al., 2015). Although their SBP vs. age results were similar, these studies showed a discrepancy in diastolic blood pressure (DBP) across a 65 year time course. The study of predominantly western European and North American subjects demonstrated a decline of DBP starting around 38 years of age in women and around 48 years of age in men (Wright et al., 2011). In the study of Iranian subjects, DBP continued to rise between the ages of 25 and 69 (Hosseini et al., 2015). Other studies have also linked blood pressure variations between individuals to age (Lee et al., 1966; Jorde et al., 1986; Nardo et al., 1999; Wu et al., 2008; Joyner et al., 2015).

With respect to gender, DBP was higher in men than women in two large studies of predominantly western European and North American subjects (Whitlock et al., 2009; Wright et al., 2011), while in the study of Iranian subjects, there was no significant gender difference in DBP (Hosseini et al., 2015). In addition to autonomic activity, inter-individual blood pressure differences between healthy subjects whose arterial pressure lay in the normal range, has been linked to gender (Wu et al., 2008; Xu et al., 2013; Joyner et al., 2015). However, an interaction between gender and autonomic activity complicates this factor: Gender differences in the time course of the cardiovascular response to orthostatic challenge suggest that men show an earlier sympathetic vascular response than women who demonstrate a more prolonged vagal restraint of cardiac activity early in the response to orthostatic challenge (Evans et al., 2001; Reulecke et al., 2016; Sarafian and Miles-Chan, 2016).

Ethnicity also appears to play a role in blood pressure differences. In the Wright study, both SBP and DBP were higher in non-Hispanic, black subjects than in non-Hispanic white and in Hispanic subjects (Wright et al., 2011). This difference may be an indicator that ethnicity might also have played a role in the DBP differences seen in western European/north American vs. Iranian subjects (Wright et al., 2011; Hosseini et al., 2015).

With respect to interpersonal differences, body mass index (BMI) has been the variable most widely used to explore relationships to blood pressure (Nardo et al., 1999). A study of 894,576 people determined an approximately linear relationship between BMI and blood pressure with an increase of SBP of ~5 mmHg and DBP of ~4 mmHg for every 5 units (kg/m^2^) of BMI (Whitlock et al., 2009). Other studies have reported scaling by height (Arvedsen et al., 2012), by weight (Whyte, 1959; Miall et al., 1968; Jorde et al., 1986), and by genetic makeup (Xu et al., 2013).

In the cardiology literature, stroke volume (SV), CO, and TPR have been normalized by body surface area (BSA) and used to describe gender etc. responses to stress (Shoemaker et al., 2001). An effort has also been made in the cardiology literature to assess cardiovascular disease incidence by height (Paajanen et al., 2010).

In contrast to cardiology literature though, most physiologic studies of healthy adults do not scale cardiovascular variables, with notable exceptions (Arvedsen et al., 2012; Joyner et al., 2015; Sarafian and Miles-Chan, 2016). When used, scaling is mostly limited to body mass index (BMI), BSA and height, with few using weight alone as a scaling factor. In total, the underlying physiological basis for much of the between-subject variability in arterial blood pressure (BP) remains unexplained in both clinical and healthy populations.

From earliest studies (Hill and Barnard, 1897), controversy concerning sources of between- subject variability in blood pressure when supine and in response to assuming the upright posture have been debated. The controversy and the relative roles of passive gravitational effects and reflex adjustments have been comprehensively reviewed (Rowell, 1983). In general, the time course of orthostatic events upon standing or head up tilt begins with an initial drop in blood pressure attributable to the immediate effects of gravity, resulting in peripheral blood pooling that “starves” the heart of venous return. This drop in pressure is countered by reflex regulatory responses: decreased cardiac parasympathetic influence, followed by increased sympathetic and hormonal activities that act over differing time courses with the result that the heart and peripheral vasculature respond and return blood pressure to a steady state, near to that of resting levels (Rowell, 1983; Fessel and Robertson, 2006). This steady state that includes reflex responses, is used to characterize a person as having orthostatic hypotension (>20 mmHg decrease), orthostatic hypertension (>20 mmHg increase) or a “normal” (between 19 mmHg increase or 19 mmHg decrease) response to standing (Rowell, 1983; Fessel and Robertson, 2006; Yatsuya et al., 2011). Orthostatic hypertension has been ascribed to hyperactive compensatory reflex activity while orthostatic hypotension reflects an inadequate compensatory reflex response (Fessel and Robertson, 2006).

The intricacies of variability contributed by these factors alone and interacting with each other, may account for the relative obscurity of the relationship of blood pressure to weight and height in adults (Whyte, 1959; Arvedsen et al., 2012; Joyner et al., 2015; Sarafian and Miles-Chan, 2016), even though positive correlations between blood pressure and body size are well documented in children and adolescents (Gillum et al., 1982; Akahoshi et al., 1996; Patel et al., 2009; Fujita et al., 2010).

More important is the fact that few studies detail cardiac and peripheral vascular components of inter-individual blood pressure variability. The present study was conducted to obtain quantitative information concerning gender, height, weight, BSA and BMI contributions to blood pressure and its cardiac output and vascular resistance components from healthy, young men and women at supine rest and during the steady state response to standing. We hypothesized that significant variability in blood pressure at rest and standing arises from underlying relationships linking SV, CO and TPR with body size (height, weight, BSA and/or BMI).

## Materials and methods

### Ethical approval

All subjects gave written informed consent to the experimental protocol, approved by the NASA Johnson Space Center and University of Kentucky Institutional Review Boards. All procedures were performed in accordance with the ethical standards of the 1964 Helsinki Declaration and its later amendments.

### Study population

Thirty-four young adults (19 men and 15 women, 8 Asian, 1 Hispanic, and 25 white) participated in 39 studies, conducted over three years. These original studies were designed to document cardiovascular effects of lower body positive pressure (LBPP) applied to subjects in a standing position (Evans et al., 2013; Kostas et al., 2014; Zhang et al., 2014). Postural controls for each of those studies consisted of data segments collected during the last 10 min of a 30-min supine control period and across 10 min of standing (without LBPP). The postural control data from these three studies were combined to give the data presented here. Demographics for these subjects are given in Table 1.

**Table 1:** Demographic features of study group.

	**Age (year)**	**Height (cm)**	**Weight (kg)**	**BMI**	**BSA**
Male (*n* = 19)	25.5 ± 5.6	176.9 ± 10.7	80.8 ± 19.4	25.1 ± 4.7	2.0 ± 0.3
Female (*n* = 15)	25.9 ± 4.1	162.3 ± 3.8	61.3 ± 8.9	23.2 ± 3.5	1.7 ± 0.1

*Data are expressed as mean ± S.D*.

### Study protocol

The initial visit consisted of a physical examination, which included a 12 lead ECG, a medical history and familiarization with study personnel, equipment and test procedures. Approximately 10 days later: following a light breakfast, the subject's age, weight and exercise history were recorded, and Neoprene shorts (needed for the LBPP chamber used in the original three studies) of the appropriate size were donned. Subjects were supine ~30 min for instrumentation placement to monitor heart rate (HR; Space Labs) and continuous pulsatile blood pressure (BP; Portapres, Finapres Medical Systems, the Netherlands). Brachial artery pressure was measured manually at each position using a cuff (UA-767, A&D Medical, Milpitas, CA) placed around the upper arm for calibration of the Portapres. Mean blood pressure was calculated from diastolic pressure plus 1/3 pulse pressure. Doppler ultrasound (Philips CX50) was used to obtain indexes of stroke volume and cardiac output. Data collection consisted of 6–7 min of echocardiography followed by three min collection of other variables. Manual BP was recorded during the last 3 min periods of supine rest and standing. Manual BP data are reported, while continuous BP data were examined for verification of results. Supine preceded standing data collection in all cases. The stand test was terminated immediately if symptoms of orthostatic hypotension developed (systolic blood pressure below 70 mmHg, HR dropped more than 20 beats per minute, or a subject reported lightheadedness, dizziness or nausea). Data from presyncopal subjects (*n* = 2) are not included in the cardiovascular results. Room temperature was maintained between 22.5 and 23.5°C. Body Mass Index was calculated from body weight/height^2^ and Body Surface Area was estimated using the square root of ((height × weight)/3600), (Mosteller, 1987).

### Data acquisition and analysis

Digital data were collected using computer acquisition software (WinDAQ, DATAQ Instruments, Akron, OH) at 1,000 Hz with subsequent analysis using MATLAB (Mathworks, Natick, MA). For the present study, we are reporting manual blood pressures and peripheral vascular resistance calculated from manually measured blood pressure. One reading of blood pressure was taken at the end of 7–10 min each at supine and standing body positions. Doppler heart images were stored for offline analysis (ProSolv w 3.0, Problem Solving Concepts, Inc., Indianapolis, IN). Images from at least three cardiac cycles for each minute were independently analyzed by two experienced, registered sonographers. Stroke volume (annulus cross sectional area × velocity time integral), cardiac output (stroke volume × heart rate), and total peripheral resistance (mean arterial pressure/cardiac output) were calculated. Group averaged data are reported as mean ± SD.

### Statistical analysis

Multiple linear regressions were performed to quantify relationships between response variables and body size measurements taken from each subject. Pearson product moment correlation and p values estimating the significance of each slope with respect to zero slope were also determined for each variable. In addition, for each dependent variable, at supine, standing and change from supine, variable selection by backwards elimination was enacted on the independent variables of gender, weight, height, BMI, BSA and all second order interactions. The cutoff level for elimination at each step was set at 0.1. Some marginal effects plots are shown to indicate the effect of the predictor on the response when the other predictor was held at its mean. Differences in slopes and intercepts were tested by regressing (DBP, SBP and MBP) blood pressure type on gender, weight (or height), and the interaction of gender by weight (or height). We then examine the significance of the interaction to test for the equality of slopes, and the significance of gender for different intercepts. All statistical analyses were performed in SAS (SAS Institute, Cary NC), with all plots constructed in R.

## Results

Table 2 gives Pearson product moment correlations (r) for four indexes of body size (weight, height, BSA, and BMI) with diastolic, systolic and mean blood pressures, stroke volume, cardiac output and total peripheral resistance, at supine rest, at standing and change from supine, for the total group of subjects (men's and women's data combined). Also given are the respective p values for each correlation and the slope of the relationship. Heart rate is not included in this table, since there was no significant correlation of HR with any index of body size at supine, standing or change from supine. Figures and multiple stepwise regression results (*r*^2^ and *p*-values, Table 3) present these findings individually and in graphical form.

**Table 2:** Pearson product-moment correlations (r) and respective *p*-values for relationships between physiological variables (diastolic blood pressure, DBP, systolic blood pressure, SBP, mean blood pressure, MBP, stroke volume, SV, cardiac output, CO and total peripheral resistance, TPR and four indexes of body size for the group of 34 subjects, (15 women and 19 men).

**Condition**	**Supine**			**Stand**			**Change**			**Supine**			**Stand**			**Change**		
***r*, *p*, slope**	***r***	***p***	**Slope**	***r***	***p***	**Slope**	***r***	***p***	**Slope**	***r***	***p***	**Slope**	***r***	***p***	**Slope**	***r***	***p***	**Slope**
**Variable**	**DBP**									**SBP**								
Weight	0.06	0.746	0.02	0.50	0.002	0.27	0.49	0.002	0.25	0.59	0.0001	0.40	0.65	< 0.0001	0.57	0.30	0.073	0.16
Height	−0.14	0.417	−0.10	0.42	0.011	0.36	0.56	0.0003	0.46	0.54	0.001	0.59	0.58	0.0002	0.82	0.26	0.125	0.23
BMI	0.18	0.298	0.34	0.38	0.019	0.89	0.25	0.132	0.55	0.43	0.009	1.23	0.48	0.002	1.8	0.24	0.152	0.57
BSA	0.001	0.995	0.03	0.50	0.002	17.8	0.53	0.001	17.7	0.60	< 0.0001	26.80	0.67	< 0.0001	38.2	0.31	0.059	11.4
**Variable**	**SV**									**MBP**								
Weight	0.50	0.002	0.560	0.32	0.051	0.25	−0.42	0.010	−0.31	0.34	0.042	0.15	0.63	< 0.0001	0.37	0.46	0.005	0.22
Height	0.63	< 0.0001	1.15	0.41	0.012	0.51	−0.54	0.001	−0.64	0.18	0.289	0.13	0.54	0.001	0.51	0.49	0.002	0.39
BMI	0.25	0.144	1.19	0.16	0.332	0.54	−0.20	0.229	−0.65	0.33	0.044	0.64	0.47	0.003	1.19	0.27	0.107	0.56
BSA	0.56	< 0.0003	41.7	0.36	0.027	18.4	−0.48	0.003	−23.4	0.31	0.066	9	0.63	< 0.0001	24.6	0.49	0.002	15.6
**Variable**	**CO**									**TPR**								
Weight	0.43	0.009	0.034	0.29	0.09	0.016	−0.36	0.028	−0.018	−0.27	0.108	−0.082	−0.02	0.901	−0.007	0.37	0.023	0.075
Height	0.48	0.003	0.062	0.36	0.03	0.032	−0.36	0.028	−0.029	−0.39	0.017	−0.192	−0.15	0.387	−0.077	0.36	0.030	0.115
BMI	0.25	0.138	0.085	0.15	0.38	0.035	−0.23	0.165	−0.05	−0.09	0.585	−0.121	0.07	0.699	0.091	0.25	0.141	0.212
BSA	0.46	0.004	2.4	0.32	0.06	1.17	−0.38	0.021	−1.24	−0.31	0.060	−6.24	−0.05	0.751	−1.152	0.39	0.018	5.085

**Table 3:** Stepwise multiple regression analysis results showing *r*^2^, adjusted *r*^2^ model *p*-values and coefficients for the response variables cited in this project.

		**Predictors**		**Adj *r*^2^**	**Model *p* values**	**Intercept**	**Coefficient weight**	**Coefficient height**	**Coefficient gender**	Coeff. Gen^*^Ht
	**Response**	**Selected**	***r*^2^**							
	SBP	gen, wt	0.45	0.42	< 0.0001	92.7 (6.66)	0.27 (0.10)	^*^	9.35 (3.72)	^*^
Supine	DBP	Intercept	0	0	< 0.0001	66.9 (1.4)	^*^	^*^	^*^	^*^
	SV	gen, ht	0.44	0.41	< 0.0001	−167.7 (51.7)	^*^	1.5 (0.32)	−11.86 (7.21)	^*^
	CO	gen, ht	0.29	0.25	0.003	−9.88 (4.1)	^*^	0.09 (0.03)	−1.01 (0.58)	^*^
	TPR	gen, ht	0.27	0.23	0.0044	74.0 (15.9)	^*^	−0.35 (0.10)	5.25 (2.21)	^*^
Stand	SBP	gen, wt	0.5	0.47	< 0.0001	81.3 (8.2)	0.42 (0.12)	^*^	10.39 (4.56)	^*^
	DBP	gen. wt	0.3	0.25	0.0026	58.6 (6.1)	0.20 (0.09)	^*^	4.77 (3.38)	^*^
Change	SBP	Weight	0.09	0.06	0.073	−11.9 (6.7)	0.17 (0.09)	^*^	^*^	^*^
	DBP	Height	0.32	0.3	0.0003	−70.1 (19.8)	^*^	0.46 (0.12)	^*^	^*^
	SV	Height	0.29	0.27	0.0007	84.1(29.5)	^*^	−0.64 (0.17)	^*^	^*^
	CO	Weight	0.13	0.11	0.0284	0.4 (0.6)	−0.02 (0.01)	^*^	^*^	^*^
	TPR	Gender	0.18	0.15	0.0096	2.6 (0.85)	^*^	^*^	3.08 (1.13)	^*^

*Data are shown at supine, standing and change from supine and include the predictors for each model*,

* *Denotes non-significant. The values in the parentheses indicate standard errors of coefficient and intercept estimate*.

**Figure 1:**
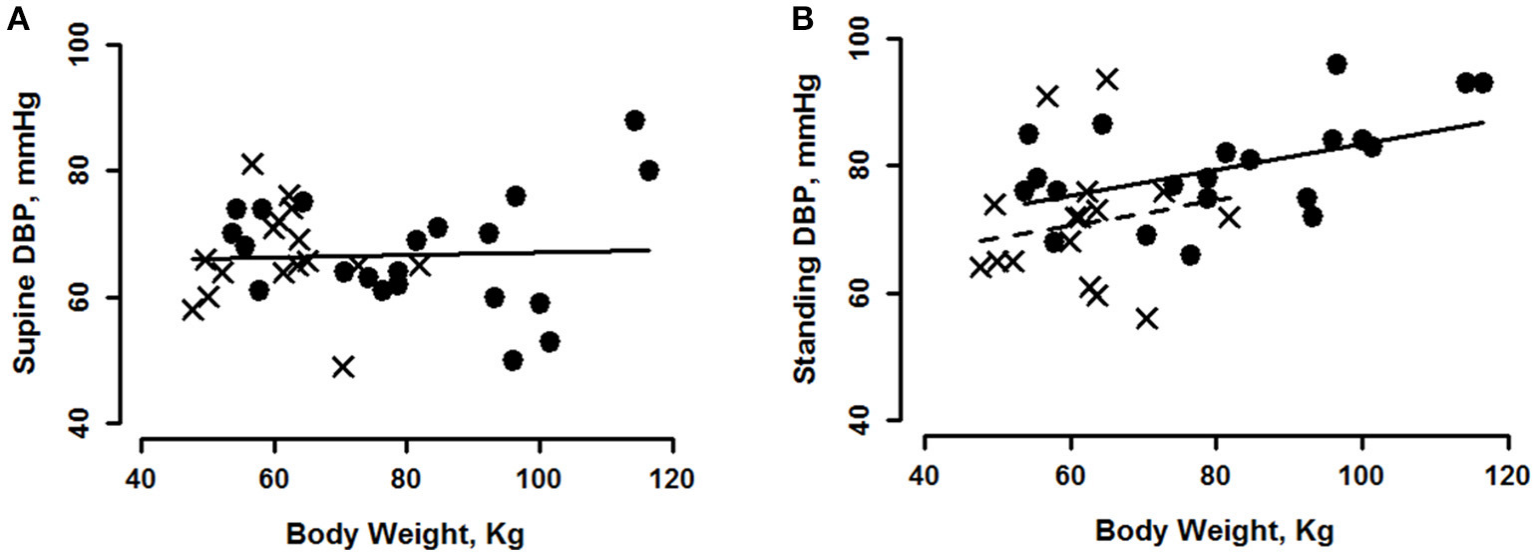
Diastolic blood pressure (DBP) plotted as a function of body weight for young men (circles) and women (crosses) at supine rest **(A)**, regression is shown for pooled data. During minutes 7–10 of standing **(B)**, regression of men's DBP on weight is shown with a solid line and women's with a dashed line. See Tables 2, 3 for statistically significant effects.

At supine, there was no indication that DBP was related to body weight, height, BMI or BSA, Tables 2, 3. In marked contrast, data taken after standing for 7–10 min indicated a significant relationship between steady state DBP and each index of body size, Table 2. The relationship between DBP and body weight is shown at supine (in Figure 1A) and at standing (Figure 1B). For supine, there was no gender effect (Table 3) and therefore men's and women's data were pooled to give a single regression line. For standing (Figure 1B and Table 3), there were weight and gender effects, but no interaction, indicating that women (crosses, dashed line) had lower values than men (circles, solid line) but followed the same regression slope. In addition to being different from 0, slopes were also different between supine and standing (*p* < 0.0004). Continuous blood pressure recordings (Portapres) gave comparable results, not shown.

**Figure 2:**
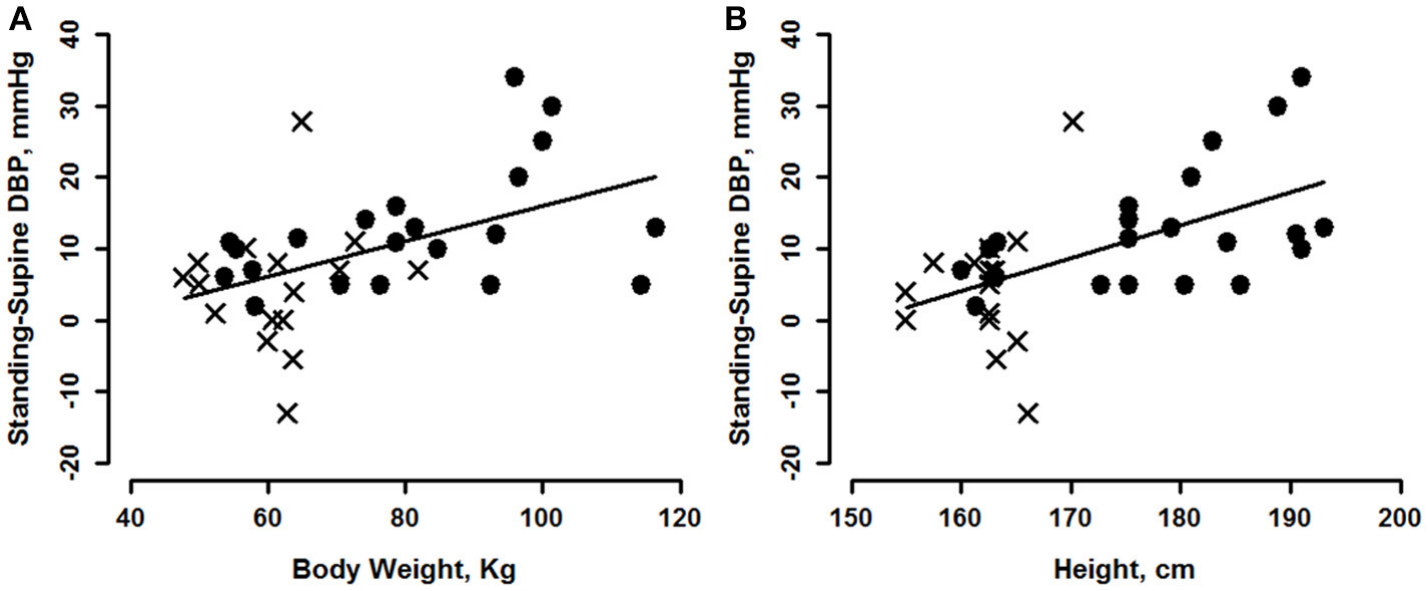
Change of diastolic blood pressure (DBP) from supine, vs. body weight **(A)** and height **(B)** for men (circles) and women (crosses). See Tables 2, 3 for statistically significant effects.

The magnitude of the change in DBP going from supine to standing provides an explanation for the difference in slopes between the two body positions. The magnitude of the increase in DBP evoked by standing was greater for subjects of greater body weight (Figure 2A), greater height (Figure 2B) or greater BSA (Table 2); the relationship with BMI was not significant. For each of these variables, there was no gender effect, so gender data were pooled. The slopes of DBP change (i.e., difference between supine and standing) vs. body weight and height (taken from Table 2), indicated that, upon standing, DBP increased ~2.5 mmHg for each 10 kg of weight, and 4.6 mmHg for each 10 cm of height in this group. These differences account for an ~17.2 mmHg greater increase in DBP for the heaviest vs. lightest of our subjects and an ~17.5 mmHg greater increase for the tallest vs. shortest of our subjects upon standing.

**Figure 3:**
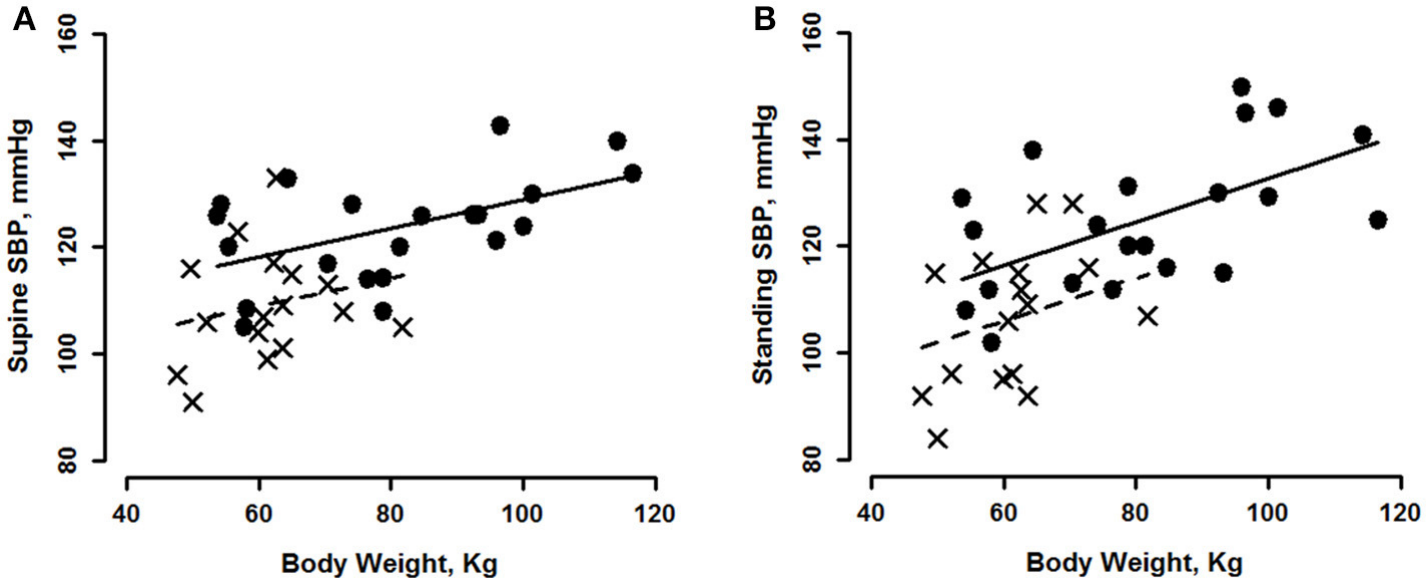
Systolic blood pressure (SBP) shown as a function of body weight for young men (circles, solid line) and women (crosses, dashed line) during minutes 7–10 of supine rest **(A)** and standing **(B)**. See Tables 2, 3 for statistically significant effects.

The relationships of SBP to indexes of body size differ from those of DBP: positive correlations of SBP with all indexes of body size were significant for both supine and standing, (Table 2). The regression relationships between SBP and weight are shown in Figure 3A for supine and in Figure 3B for standing. For SBP, there were significant gender effects, but no interactions (Table 3), so the data in Figure 3 show the same slope for men and women. Slopes from Table 2 indicate an ~4 mmHg SBP higher value for each 10 kg of body weight and ~5.9 mmHg for each 10 cm height, when supine and ~5.9 mmHg/10 kg weight and ~8.2 mmHg/10 cm height, when standing. The change in SBP going from supine to standing correlated with weight, when holding all other body size predictors constant, thus weight had the greatest influence on changes in SBP, Table 3.

The relationships between mean arterial blood pressure (MBP) and body size have a resemblance to both systolic and diastolic pressures (Table 2). When supine, significant correlations of MBP with weight and BMI resulted from systolic pressure contributions to mean pressure (since there were no significant correlations of DBP with any index of body size when supine). Standing MBP was positively related to all indexes of body size as were SBP and DBP while change in MBP was dominated by DBP as both correlated positively with height, weight and BSA.

**Figure 4:**
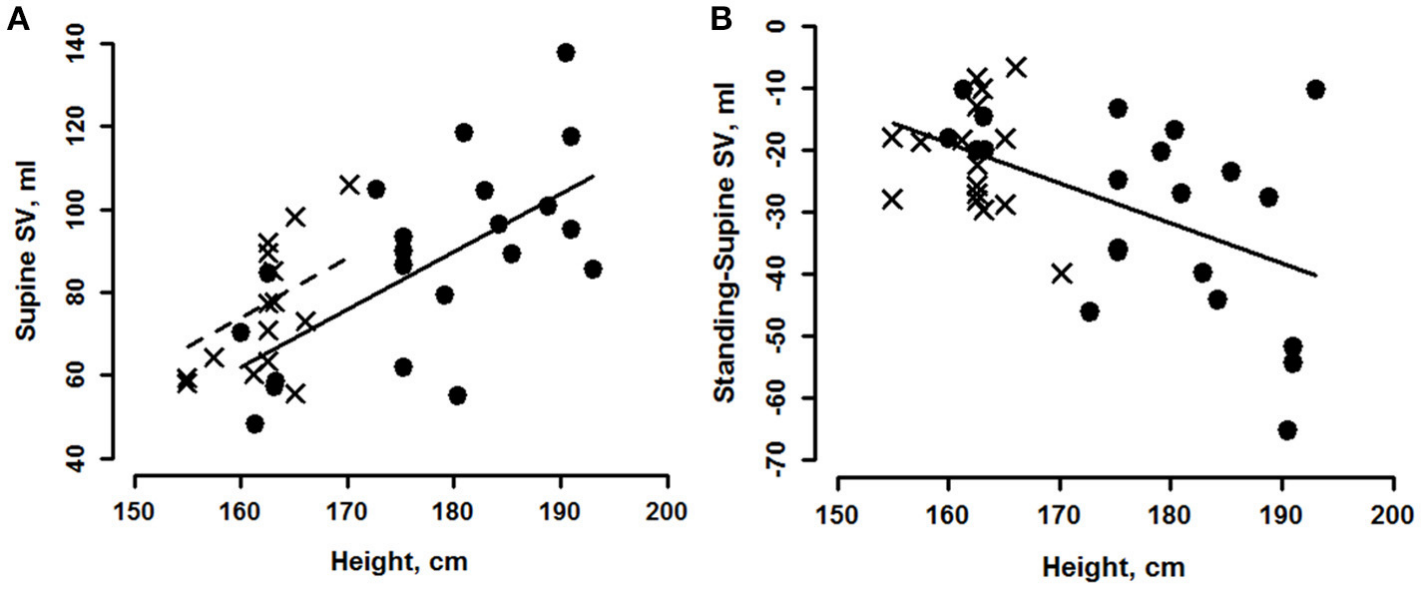
Marginal effects plot of supine stroke volume (SV, **A**) vs. height for young men (circles, solid line) and women (crosses, dashed line). Change from supine **(B)** vs. height for young men (circles) and women (crosses). See Tables 2, 3 for statistically significant effects.

Doppler measures of stroke volume (SV) for these subjects, indicated that supine values of SV, as well as changes in SV upon standing, correlated strongly with height, weight and BSA, but not with BMI, Table 2. There were significant gender and height effects, but no interactions, allowing us to state that the taller the person, the greater was their supine resting stroke volume. The relationship between SV and height is shown in Figure 4A for supine. We estimate that supine SV averaged ~11.5 ml greater, for 10 centimeters of height. Correlations of standing SV with height, weight and BSA were weaker than they were for supine and are not plotted, but correlations of indexes of body size with the change in stroke volume, were strong, Tables 2, 3. The relationship between height and change in SV upon standing is shown in Figure 4B. Again, there was no gender interaction so data are pooled. The taller the person, the greater their decline in stroke volume on standing, approximately 6.4 ml greater, for a 10 cm increase in height, Table 2 and Figure 4B.

The increase (compared to supine) in heart rate at 10 min of standing was 17.6 ± 5.6 beats per min. Neither supine heart rate, standing heart rate, or change upon standing, correlated with body weight, height, BSA or BMI. The largest correlation coefficient of HR with any of these variables was 0.15, not shown.

Cardiac output in the supine position, correlated with weight, height and BSA, but not with BMI, (Table 2). The relationship between supine CO and height was similar to, but weaker than, the relationship between SV and height and is not plotted. The change from supine to standing also correlated with weight, height and BSA, Table 2. The relationship between the change in CO and height was again similar to, but more moderate than, the SV response and is not plotted. As with SV, the taller the person, the greater their supine and standing CO, and decrease of CO upon standing.

**Figure 5:**
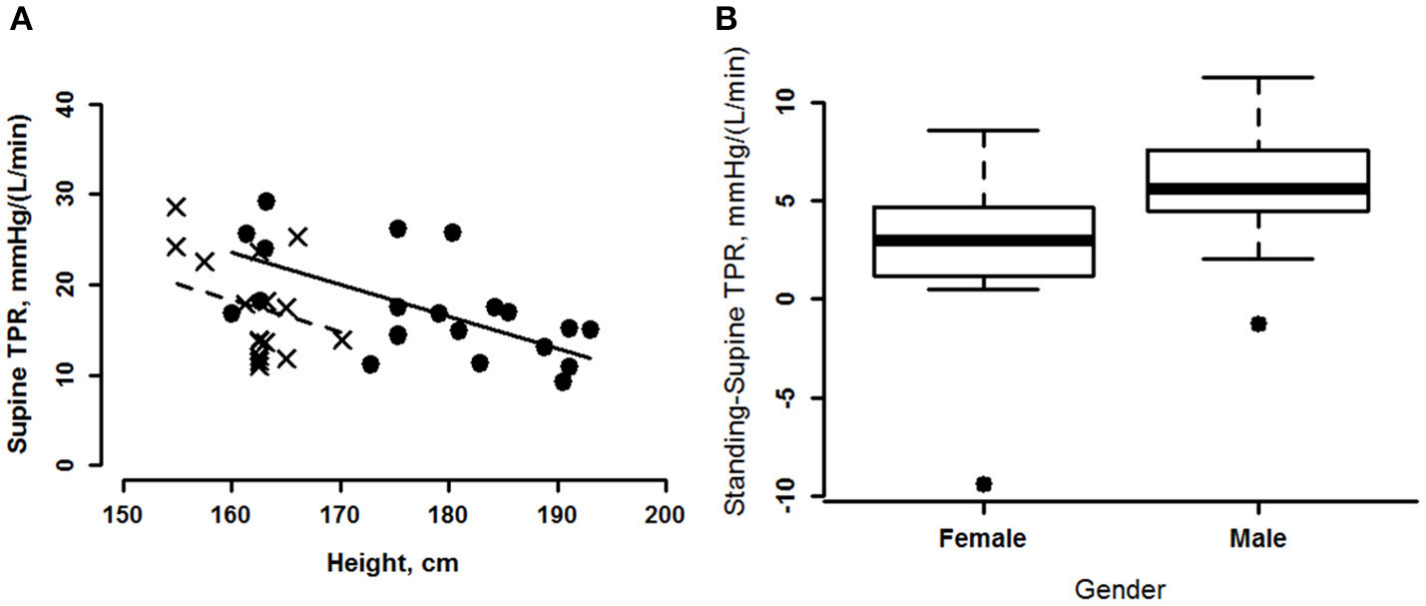
**(A)** Marginal effects plot of supine total peripheral resistance (TPR) plotted as a function of height for men (circles, solid line) and women (crosses, dashed line). **(B)** TPR change from supine for men and women shown as boxplots. The horizontal line in the rectangle shows the median. The central rectangle spans the interquartile range. The whiskers represent minimum and maximum values. The single data points on both the men's and women's plots, indicate outliers.

Supine values of total peripheral resistance (TPR) correlated negatively with height (Table 2 and Figure 5A) with significant gender and height effects, but no interaction (Table 3). On standing, the correlation between height, weight and BSA reversed to a positive correlation (Table 2) with a slight, but significantly greater increase in women's, compared to men's, TPR (Figure 5B).

**Figure 6:**
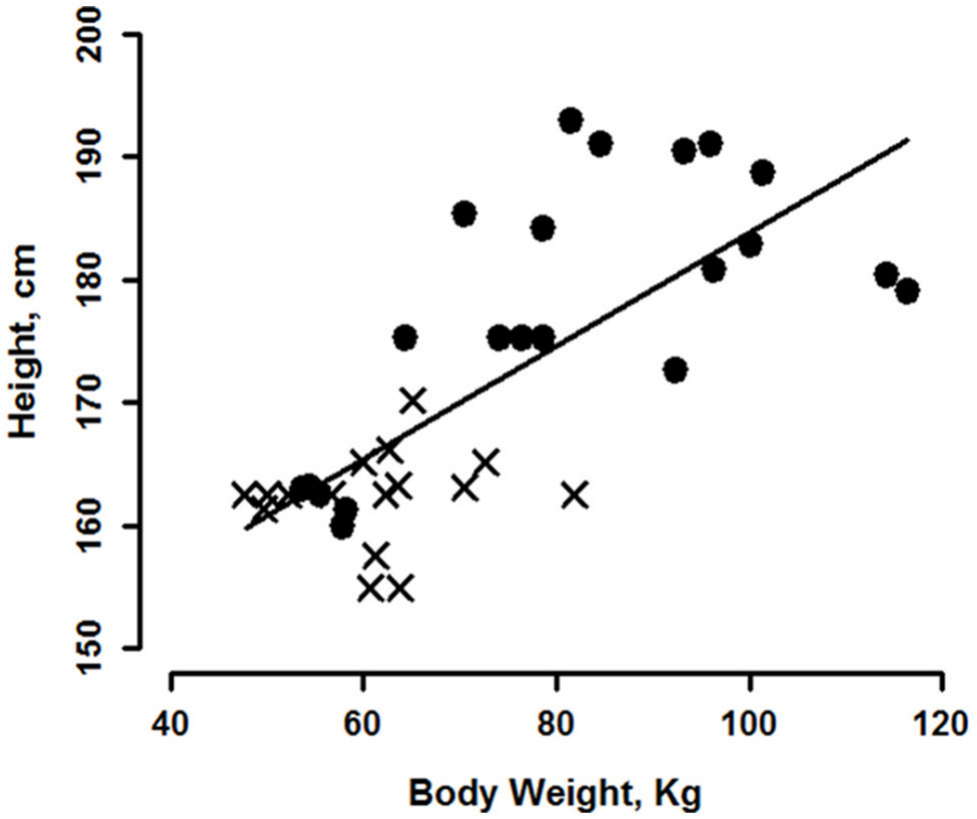
Regression of subject height on weight for 22 men (circles) and 16 women (crosses). Regression line is for the group of men and women combined.

As one would expect, body height and weight were positively correlated, the greater a person's height, the greater was their weight, with the correlation more apparent in men than women, Figure 6. It is therefore not surprising that the variables we report correlate with weight, height and BSA though we note that correlations of most variables with BMI were not significant. Our data do indicate that blood pressure variables correlated more highly with weight while stroke volume, cardiac output and total peripheral resistance correlated more highly with height. For all cardiovascular variables, women and men did not differ in terms of their regression slopes; this group of women populated the lower end of the height and weight scales. However, the relationships we are reporting may not be the case for taller women; we did not have females in the higher weight/height ranges. This was the explanation proposed in a recent study reporting correlations between blood pressures and height that were readily apparent in their male, but not female, subjects (Arvedsen et al., 2012).

## Discussion

We observed significant positive correlations between body weight, height, BMI, and BSA with steady state, diastolic blood pressure when these young men and women were standing. Conversely, we found no such correlation with DBP in the supine state. Moreover, the more a person weighed, the taller s/he was, or the greater their body surface area, the greater their increase in diastolic pressure upon standing. In contrast, systolic pressure showed strong correlations with weight, height, BSA and BMI both supine and standing, but no significant correlations between postural changes in SBP with any index of body size. Most important, we found that inter-individual variabilities in cardiac output, stroke volume and vascular resistance related to body size, provide mechanistic explanations for the associated inter-subject variability in blood pressure that is different in supine and standing positions.

### Cardiac and vascular contributions to blood pressure

We can now evaluate several of our blood pressure findings within the context of cardiac and vascular function. First, at supine rest, the combination of the positive correlation of cardiac output with height and the negative correlation of peripheral resistance with height, resulted in a lack of correlation of supine diastolic blood pressure with any index of body size. Second, upon standing, the combination of a continued positive correlation of CO with height and a significant correlation of the increase in TPR with height, were responsible for the positive correlation of diastolic blood pressure with height. Third, at supine, positive correlations between CO (due to SV) and all indexes of body height except BMI, led to the positive relationships between SBP and those same indexes of body size. Fourth, upon standing, the strong relationship between SBP and any index of body size resulted from the combination of positive relationships between standing SV and change in TPR, with most indexes of body size. Finally, mean blood pressure reflected the effects of systolic, more than diastolic, pressure in terms of relationships to indexes of body size. To the best of our knowledge, these are new observations of the relationship between body size and arterial pressure regulation in young adults and illustrate the importance of considering physical attributes and posture when evaluating such data. They also add a new consideration to the frequently noted association of hypertension with obesity.

Population and insurance company studies typically indicate a consistent, but weak, relationship between body weight and blood pressure; however, in most cases those correlations came from data collected in seated subjects (Miall et al., 1968). A study of 100 seated men, with blood pressures taken prior to blood donation, determined that both systolic and diastolic blood pressures correlated significantly with body weight, but not with subcutaneous fat (measured at three anatomical sites), except as that fat contributed to total body size (Whyte, 1959). A 1986 study of 618 healthy adults determined a significant, positive relationship between SBP and body size (reported as a composite of height, weight, wrist diameter, and grip strength; Jorde et al., 1986). In addition, their study determined that standing, but not supine, diastolic pressure correlated with their measure of body size. That study's blood pressure/body weight results (and the fact that they found that obesity was not a factor) support our study in that both studies found a significant correlation between body size and diastolic pressure when subjects were standing but not when supine as well as significant correlations of body size to systolic pressure both supine and standing (Jorde et al., 1986). These studies however, did not determine the cardiac output and peripheral vascular components of blood pressure.

A recent study of blood pressure regulation during postural change determined a dependence of cardiovascular variables on body height in a demographic similar to our own (Arvedsen et al., 2012). These investigators reported a two times greater stroke volume response to upper body tilting in their tallest vs. shortest subjects. The tilt portion of their study did not find a dependence of blood pressure on height, but when they looked at the results of 24 h BP monitoring in the same subjects, they did find a significant, positive relationship between blood pressure and height in males and a tendency toward the same in females. The heart rate and blood pressure differences between our study and theirs is likely due to their smaller orthostatic maneuver (half body tilt), differences in the body's response to tilt vs. standing, the direction of positional change (seated to supine), and/or differences in the timing of data sampling.

Another recent study, again demographically similar to our study, looked at adaptation to head up tilt, and determined that, at supine rest, differences in SV, HR, and TPR components of BP appeared to be more attributable to body size, in particular skeletal muscle mass and body height, than to gender (Sarafian and Miles-Chan, 2016). They further determined that, in response to orthostatic challenge, these men and women exhibited different patterns of autonomic responses that were clearly not due to anthropometry. The complexity of dissecting gender, anthropometry, and autonomic interactions in the regulation of blood pressure will not be a simple process, but results from the present study, our previous study (Evans et al., 2001) together with the Reulecke (Reulecke et al., 2016), Barantke (Barantke et al., 2008), and Sarafian studies (Sarafian and Miles-Chan, 2016), indicate that even though gender may not be a component of anthropometry-based cardiovascular measures, it becomes important in measures of autonomic activity.

### Gender

We examined all data for gender differences. Even though we found a gender interaction for height vs. weight, the women of our study fell into lower height and weight categories thereby explaining the simple gender effect seen in other variables. In addition, women's data occurred in narrower ranges of heights (15 cm [women] vs. 33 cm [men]) and weights (34 kg [women] vs. 62 kg [men]). Therefore, our study does not provide sufficient data to draw conclusions about all women of this age, and the question of gender effects remains open until larger ranges for women's heights and weights are studied. Women's and men's diastolic pressure responses to standing were similar; all men and 81% of women increased, or did not change, diastolic pressure upon standing, and the taller or heavier the person, the greater their increase in DBP upon standing. When examined separately, our men's and women's cardiovascular data exhibited the same regression slopes (Figures 1–5), with women's data clustered at the lower end of the height and weight scales, hinting that body size may have been as influential in determining the magnitude of the orthostatic blood pressure change as gender was. This finding is supported by other studies of young, healthy men and women (Dewey et al., 2008; Sarafian and Miles-Chan, 2016). The fact that the time course of sympathetic and parasympathetic responses to orthostatic challenge differs between men and women may have obscured the underlying importance of anthropometry in blood pressure regulation.

### Obesity, age, and ethnicity

Only four of our subjects were obese (i.e., BMI > 30), they were relatively young, and the ethnic makeup of our study did not contain enough subject groups to make comparisons, so that ethnicity, age and obesity were not factors in our study. Therefore, gender, height, weight, BMI and BSA were the primary independent variables of the present study.

Even though our group was limited in age, age is a known determinant of blood pressure (Wright et al., 2011; Hosseini et al., 2015). In a recent review (Joyner et al., 2015), a gender by age interaction in TPR was clearly demonstrated for muscle sympathetic nerve activity that correlated directly with peripheral vascular resistance in men at all ages but only in women after they became post-menopausal (Joyner et al., 2015). This same review also documented that sympathetic activity increased with age in both men and women with the following commentary “How best to scale the determinants of blood pressure?” (Joyner et al., 2015). These authors note that “Comparisons between men and women are confounded by differences in body size, body composition and other variables. There is no generally accepted, or universal, approach to scaling these differences….thus we generally avoid scaling….” In order to answer this very important question, additional studies in a large group of men and women using a range of ages, ethnicities, heights, and weights while monitoring as many scaling variables as possible will need to be conducted.

### Height, weight, BMI, and BSA as independent variables and scaling of factors

Even though BMI has been clearly shown to influence blood pressure (Whitlock et al., 2009), those results were based on a very large number of subjects. Our study illustrates the fact that, in a small group of subjects, BMI was not as strongly related to BP as were weight, height or BSA. This may indicate a limitation in the use of the BMI combination of height and weight, compared to use of these variables alone. Even though body mass index is the most commonly used index of obesity, its ability to define that factor has been widely questioned for many reasons (Romero-Corral et al., 2008). Starting with the definition of BMI, the use of height^2^ in the denominator is arbitrary and values for the height exponent ranging from 1.45 to 3 have been proposed. Other major objections to the use of BMI have been that it does not differentiate between muscle mass and fat mass (Romero-Corral et al., 2008; Okorodudu et al., 2010), its ability to predict health has been questioned (Romero-Corral et al., 2006), dividing lines between obese, normal, underweight and overweight categories are questionable, and other indexes may be more appropriate (MacKay, 2010). Cardiology literature demonstrates a concerted effort to determine which body size parameters best predict cardiovascular parameters, particularly blood flow (West and Brown, 2005; Dewey et al., 2008). One of these studies (Dewey et al., 2008) makes a strong claim that fat free mass is the best criteria, and failing that measure, the best parameter is height. Both studies emphasize the importance of allometric scaling of cardiovascular parameters to provide normative ranges for clinical use but controversy over the appropriate allometric exponent is widespread. In scaling for medication dosage, pharmacology literature notes the widespread use of the conservative 0.67 exponent to relate metabolic rate to body weight and then proposes the exponent 0.75 to provide a more realistic relationship (Sharma and McNeill, 2009). The review by Joyner et al. (2015), proposes to test scaling by weight to the 0.67–0.75 power as well as by unscaled (ratiometric) weight. That our data significantly correlated cardiovascular variables with height or weight on the ratiometric scale (allometric exponent = 1) indicates that further study focused on scaling factors and using a much larger group of subjects should be conducted. The fact that BMI was by far the poorest body size scaling index for blood pressure, stroke volume and peripheral vascular resistance may lie simply in the fact that it is an index of obesity and other studies have indicated that the best scaling factor for cardiovascular variables is fat free mass.

These results further emphasize the importance of evaluating many physiological phenomena within the context of body size. West and Brown, for example, report such scaling over many orders of magnitude across living forms for metabolic power, proxies for which are cardiac output or blood volume flow rate through the cardiovascular system (West and Brown, 2005). Others (Dewey et al., 2008) emphasize the critical importance of body size measurements in cardiovascular medicine. Our studies emphasize that the findings regarding the role of obesity in the etiology of hypertension must be carefully considered within the context of specific assessments of body orientation, size and mass.

The effect of aging on the relationship between body size and cardiovascular variables is not known. However, the fact that these relationships are firmly established in pediatric literature, and the present and related studies extend pediatric results to young adults, but these relationships are not commonly recognized in older adults, hints that the effect may diminish with age.

### Heart rate

Our study clearly demonstrated a lack of dependence of steady state heart rate (resting supine, after 10 min of standing or difference between supine and standing) on any of the indexes of body size. However, indexes of body size might have been a factor if assessed earlier than after 10 min of standing. This lack of contribution of heart rate leaves stroke volume as the determinant of cardiac output/body weight/height correlations reported here.

## Summary

The present study identified a significant positive correlation between diastolic blood pressure and body size (weight, height, or BSA) in healthy young adults while standing, a relationship that was not present for DBP while supine. The magnitude of the standing-induced increase in diastolic pressure was also height/weight/BSA-dependent, thereby influencing standing diastolic BP. In addition, the magnitude of the standing-induced change in cardiac output was negatively related to body size, therefore, the positive correlation between peripheral resistance increase and body size accounted for the positive correlation between standing diastolic pressure and body size. We hypothesize that this relationship reflects a greater increase in vascular sympathetic activity in larger people when they undergo orthostatic stress.

At supine rest, our subjects also demonstrated a significant positive correlation between supine systolic blood pressure and body size. The mechanism for this relationship appeared to be through a positive correlation between supine stroke volume and height, which overpowered the negative correlation between resting peripheral resistance and height to produce a positive correlation between supine SBP and all indexes of body size.

## Conclusions

In healthy, young men and women, systolic, but not diastolic, blood pressure at supine rest was positively correlated with all indexes of body size due to positive resting stroke volume and cardiac output correlations. Upon standing, the correlation between the increase of diastolic pressure with weight, height and BSA was attributable to the magnitude of peripheral vascular resistance increase that correlated with weight, height and BSA.

## Limitations

Our subjects were primarily young (24.4 ± 4.9 (SD) year) and therefore we could not reliably assess effects of age. In addition, there were too few obese subjects in the present study to characterize the relationship of variables to body weight in the obese range. Women's data lay in narrower height and weight ranges than did men's data, therefore, similarities between men's and women's results, may not hold for women whose height and/or weight lie outside these narrow ranges. Subsequently, the primary independent variables of the present study consisted of weight and height and their combination in the variable BSA, but not BMI. Four subjects participated in more than one study, but, because studies were conducted a year or more apart, and their weights were different, we decided to use both data points. All subjects wore Neoprene shorts at all stages of the study, and, even though effects of these shorts on blood pressure in response to standing or head up tilt were not large (Kostas, 2012), the study perhaps should be repeated in subjects unencumbered by the abdomen and pelvic compression these shorts impart. Protocols of the three studies were slightly different but it is unlikely that those differences contributed to variability in these subjects' postural control data. Cardiac output and manual blood pressure were measured 5–7 min apart perhaps contributing to error in the peripheral vascular resistance calculation. However, there was no significant difference in group peripheral resistance calculated this way and resistance calculated from mean blood pressure taken from continuous measures of BP during the time of cardiac output measurement.

## Author contributions

JE: Co-I of the three original studies, principal author of the present study and co-author of three preceding manuscripts, wrote, revised and approved this manuscript and agrees to be accountable for all aspects of the work. SW: Data collection, analysis and manuscript development for one of the three original studies; extensive examination and statistical testing of data used in the present manuscript. CG: Responsible for data consolidation from previously conducted studies. Responsible for plots of consolidated data, interpretation and helping draft the first versions of this manuscript. VK: Data collection, analysis and manuscript development for one of the three original studies and replotting of data used in the current manuscript. CK: PI of original studies, co-author of present ms and three preceding manuscripts. QZ: Data collection, analysis and manuscript development for one of the three original studies. ER: Statistical analysis of data included in manuscript, preparation of that portion of manuscript. MS: Responsible for all aspects of cardiac Doppler data collection/analysis for original three studies plus manuscript preparation for all four studies. DR: Data critique, drafting and writing of current manuscript.

### Conflict of interest statement

The authors declare that the research was conducted in the absence of any commercial or financial relationships that could be construed as a potential conflict of interest.

## Abbreviations

ANCOVA: Analysis of covariance
BSA: Body Surface Area
BW: body weight
BMI: Body mass index
CI: Confidence interval
CO: Cardiac output
DBP: Diastolic blood pressure
HT: Height
HR: Heart rate
LBPP: Lower body positive pressure
MBP: Mean blood pressure
MSNA: Muscle sympathetic nerve activity
SV: Stroke volume
SBP: Systolic blood pressure
TPR: Total peripheral resistance.
